# Supplementary material for: FOXP3+ regulatory T cell perturbation mediated by the IFNγ-STAT1-IFITM3 feedback loop is essential for anti-tumor immunity
Source: Nat Commun. 2024 Jan 2;15:122. doi: 10.1038/s41467-023-44391-9 (PMC10761945; doi:10.1038/s41467-023-44391-9)
Supplement: Supplementary file 1 — Supplementary Information [file 41467_2023_44391_MOESM1_ESM.pdf]

Supplementary Information

Supplementary Figure1

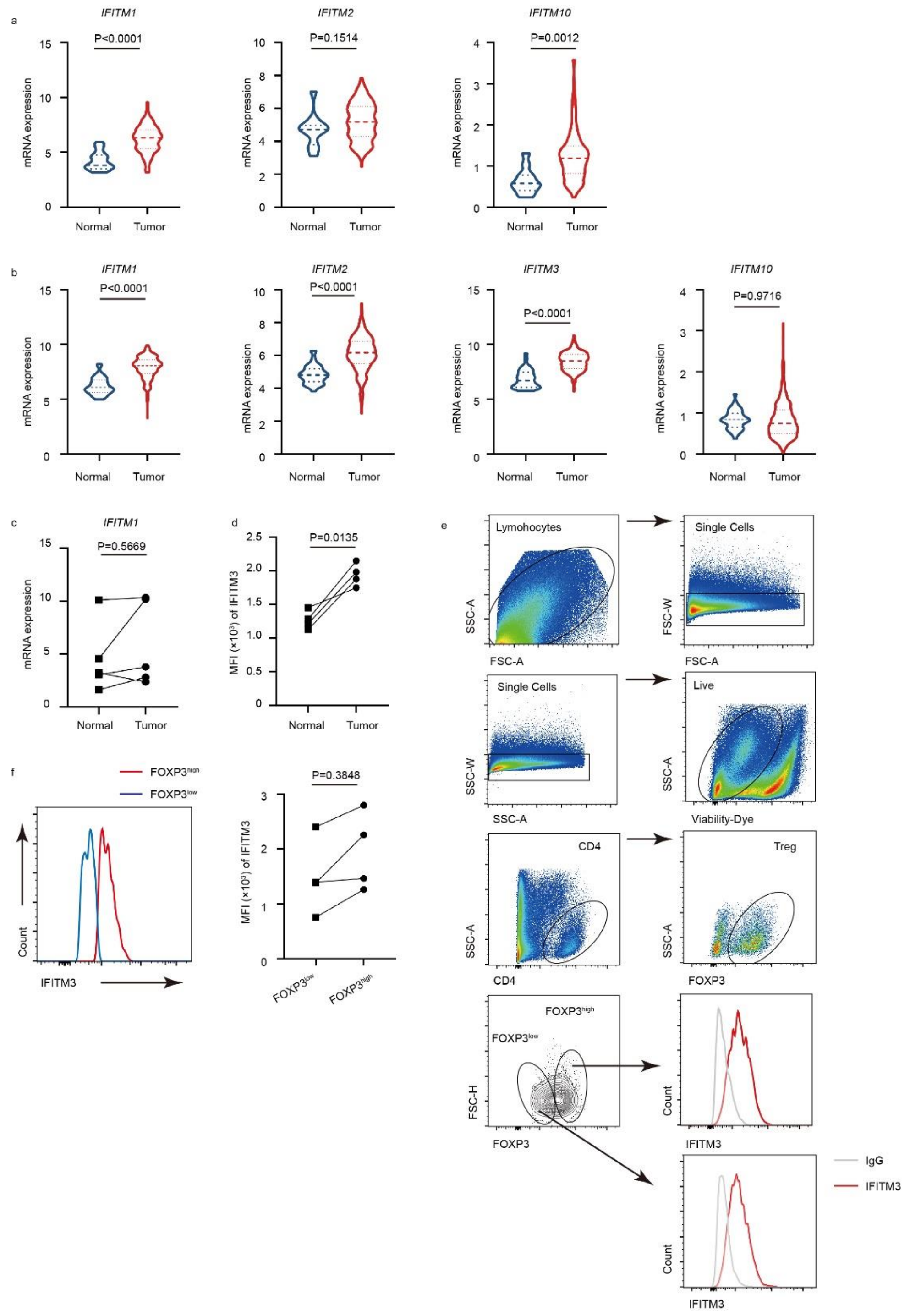

Supplementary Figure 1. IFITM3 is correlated with Treg cells in the tumor microenvironment.

(a) mRNA expression of *IFITM1*,*IFITM2*,*IFITM10* in TCGA ESCA database. (b) mRNA expression of genes in IFITM family in TCGA COAD database. (c) qRT-PCR analysis of *IFITM1* in tissue from COAD patients (n=6). (d) Histogram shows the MFI of IFITM3 and the

quantification of the MFI of IFITM3 in Treg cells from normal tissue and tumor tissue of COAD patients (n=4). (e) Flow cytometry strategy of Treg cells from the tissue of tumor patients. (f) Histogram shows the MFI of IFITM3 and the quantification of the MFI of IFITM3 in FOXP3^{high} and FOXP3^{low} tumor Treg cells. Data are represented as the mean ± SD. *P < 0.05 , **P < 0.01 and ***P < 0.001 by 2-tailed Student's t test.

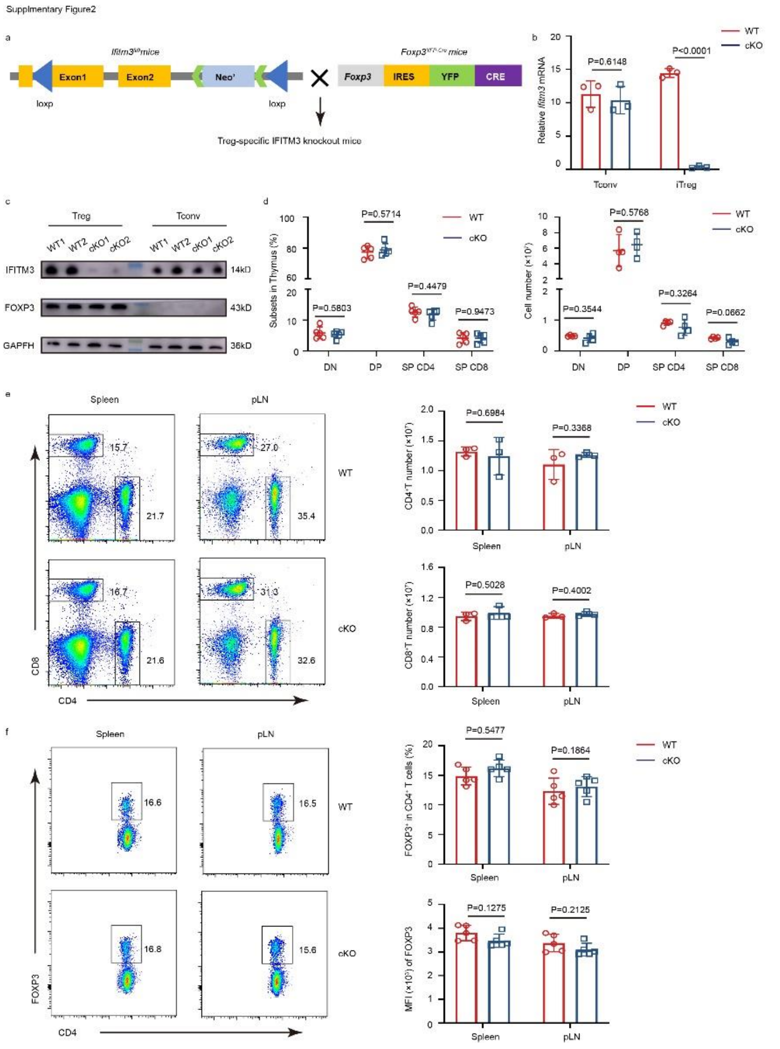

Supplementary Figure 2. IFITM3 does not influence the T cell homeostasis *in vivo*.

(a) Strategy to generate Tregs-specific IFITM3 knockout mice by crossing *Ifitm3*$^{fl/fl}$ mice with *Foxp3*^{YFPcre} mice. (b) qRT-PCR analysis of Ifitm3 expression in Treg cells and Tconv cells from WT and cKO mice. (c) Immunoblot analysis of IFITM3 expression in Treg cells and Tconv cells from WT and cKO mice. (d) Flow cytometric analysis of the percentage (n=5) and the number (n=4) of CD4^{+} T cells (SP CD4), CD8^{+} T cells (SP CD8), CD4^{+}CD8^{+} double positive (DP), and CD4^{-}CD8^{-}double negative (DN) in the thymus from WT and cKO mice. (e) Flow cytometric analysis of CD4^{+} T cells and CD8^{+} T cells in spleen and lymph nodes from WT and cKO mice (n=5). Flow cytometric analysis of CD4^{+} and CD8^{+} T cells number in spleen and

lymph nodes from WT and cKO mice (n=3). (f) Flow cytometric analysis of FOXP3$^+$ Treg cells of CD4$^+$ T cells in spleen and lymph nodes from WT and cKO mice (n=5). Data are representative of 3 independent experiments. Data are represented as the mean ± SD. *P < 0.05 , **P < 0.01 and ***P < 0.001 by 2-tailed Student's t test.

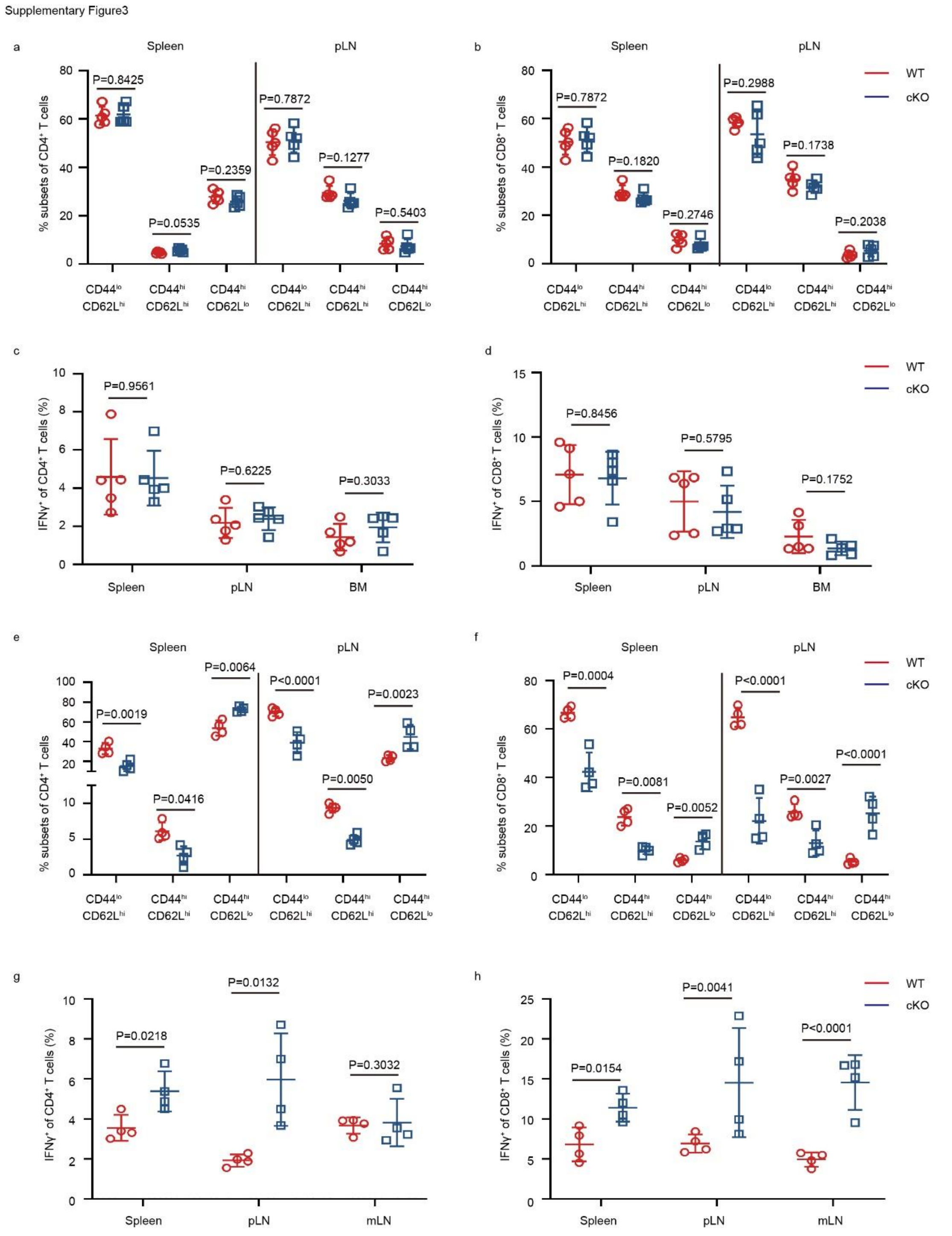

Supplementary Figure 3. IFITM3 deficiency in Treg cells causes inflammation in elder mice.

(a and b) Flow cytometric analysis of CD44 and CD62L expression in Tconv (a) and CD8$^+$ T cells (b) from WT and cKO mice (14 weeks) (n=5). (c and d) Flow cytometric analysis of IFNγ

secretion in Tconv (c) and CD8$^+$ T cells (d) from WT and cKO mice (14 weeks) (n=5). (e and f) Flow cytometric analysis of CD44 and CD62L expression in Tconv (e) and CD8$^+$ T cells (f) from WT and cKO mice (12 months) to identify the activation status of elder mice (n=4). (c and d) Flow cytometric analysis of IFNγ secretion in Tconv (g) and CD8$^+$ T cells (h) from WT and cKO mice (12 months) to identify the activation status of elder mice (n=4). Data are representative of 3 independent experiments. Data are represented as the mean ± SD. *P < 0.05 , **P < 0.01 and ***P < 0.001 by 2-tailed Student's t test.

Supplementary Figure4

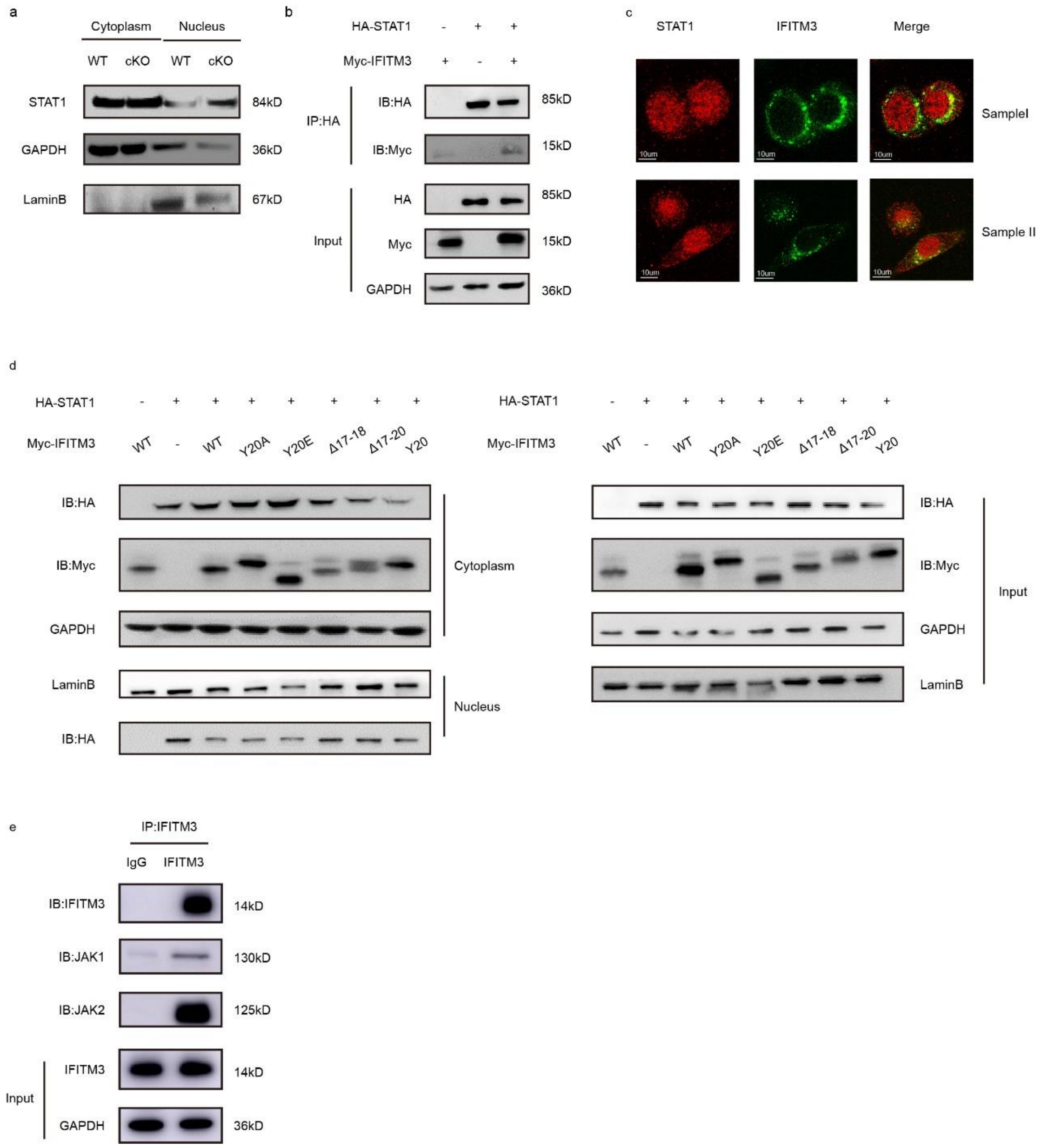

Supplementary Figure 4. IFITM3 participates in STAT1 phosphorylation.

(a) Immunoblot analysis of STAT1 in nucleus and cytoplasm extracted from WT and cKO Treg cells. (b) HEK293T cells transfected with HA-tagged STAT1 and Myc-tagged IFITM3 were

immunoprecipitated with anti-HA antibody and assessed by immunoblotting with anti-Myc. (c) Immunofluorescence analysis of STAT1 and IFITM3 correlation in Hela cells. (d) HEK293T cells were transfected with HA-tagged STAT1 and Myc-tagged IFITM3 and mutation of Myc-tagged IFITM3. Nuclear and cytoplasm extraction from HEK293T cells was then detected by immunoblotting. (e) Cell lysates of mouse Treg cells were immunoprecipitated with anti-IFITM3 antibody and assessed by immunoblotting with anti-JAK1, anti-JAK2. Experiments were independently repeated 3 times.

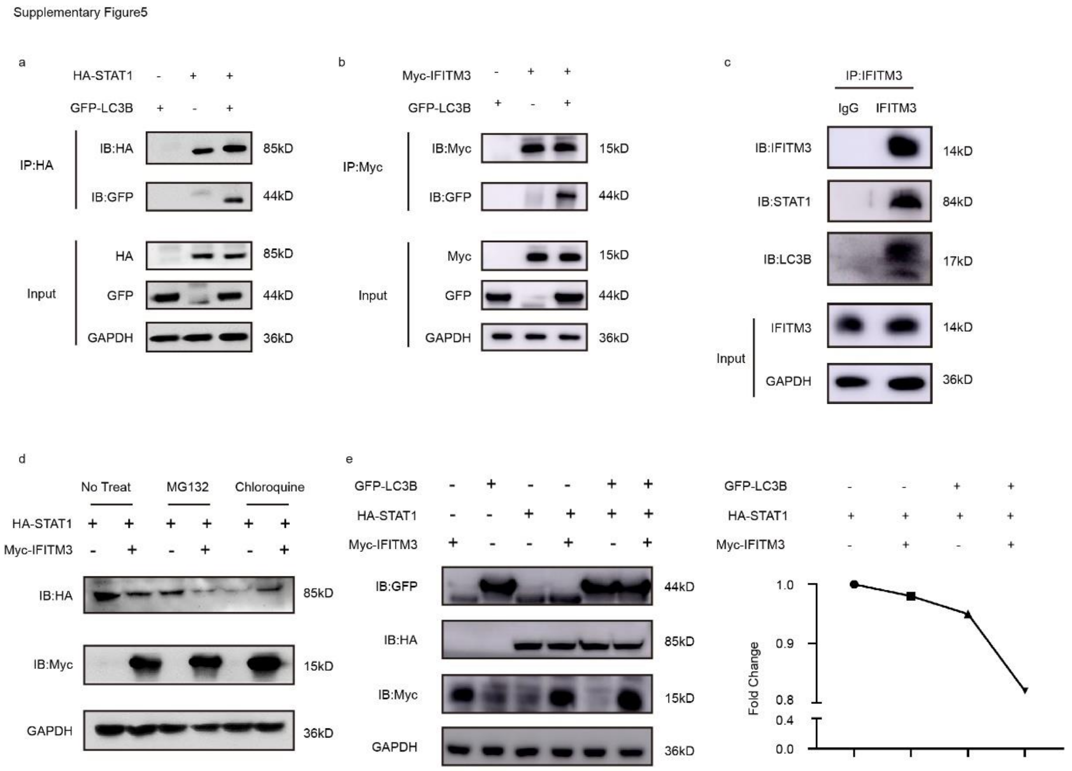

Supplementary Figure 5. IFITM3 mediates autophagic degradation of STAT1.

(a) HEK293T cells transfected with HA-tagged STAT1 and GFP-tagged LC3B were immunoprecipitated with anti-HA antibody and assessed by immunoblotting with anti-GFP. (b) HEK293T cells transfected with the Myc-tagged IFITM3 and GFP-tagged LC3B were immunoprecipitated with anti-Myc antibody and assessed by immunoblotting with anti-GFP. (c) Cell lysates of mouse Treg cells were immunoprecipitated with anti-IFITM3 antibody and assessed by immunoblotting with anti-STAT1, and anti-LC3B. (d) HEK293T cells were transfected with HA-tagged STAT1 and Myc-tagged IFITM3 and treated with MG132 or chloroquine for 6 h, then an immunoblot analysis was performed. (e) HEK293T cells were transfected with HA-tagged STAT1, Myc-tagged IFITM3, and GFP-tagged LC3B and then an immunoblot analysis was performed. The statistical data displayed the fold change of HA-STAT1 expression to lane 3 in the left panel. Experiments were independently repeated 3 times.

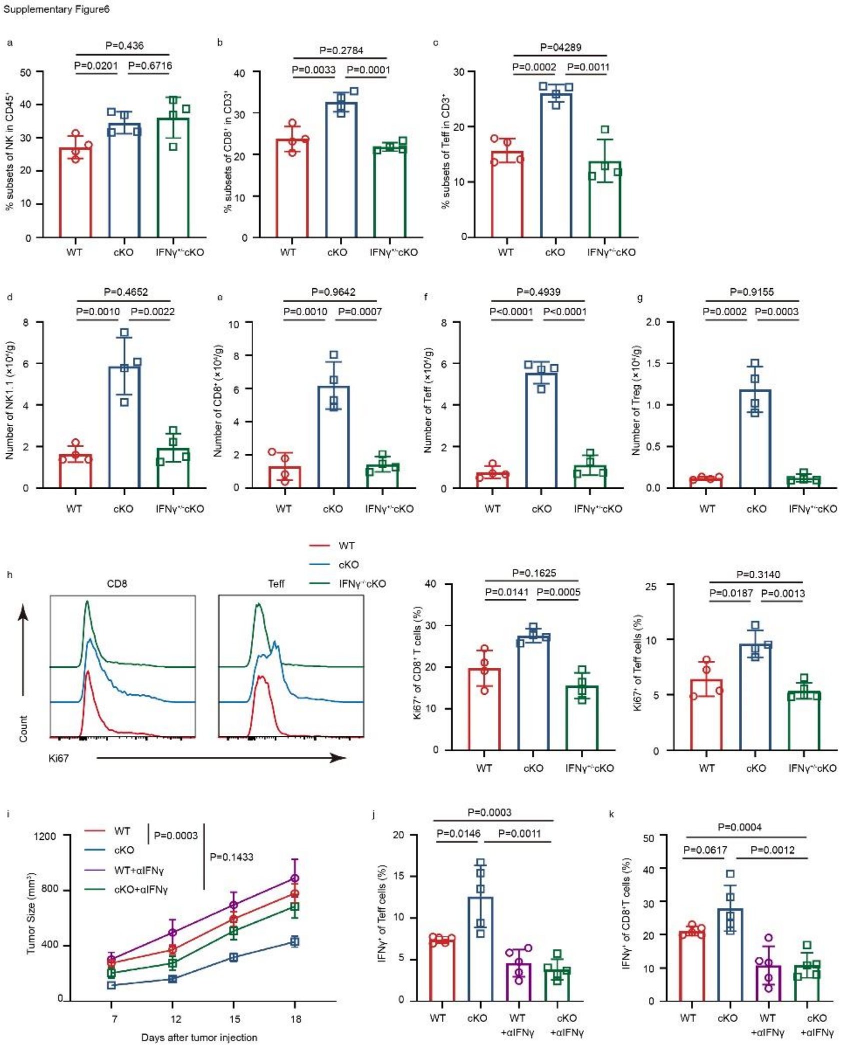

Supplementary Figure 6. The regulation of IFITM3 on tumor-infiltrating Treg cells is dependent on IFNγ.

(a-d) Percentage of NK cells (a), $CD8^+$ cells (b), and Teff cells (c) in tumor of WT, cKO, and IFNγ $^{+/-}$cKO mice injected s.c. with MC38 murine colon cancer cells (day23, n=4). (d-g) Cell number of NK cells (d), $CD8^+$ cells (e), Teff cells (f), and Treg cells (g) in tumor of WT, cKO, and IFNγ $^{+/-}$cKO mice injected s.c. with MC38 murine colon cancer cells (day23, n=4). (h) Histogram shows the MFI of Ki67 and the quantification of the MFI of Ki67 in $CD8^+$ T cells and $CD4^+$ T cells in tumors of WT, cKO, and IFNγ $^{+/-}$cKO mice injected s.c. with MC38 murine colon cancer cells(day23, n=4). (i) Tumor size of WT and cKO mice MC38 tumor model injected with anti-IFNγ (day20, n=5). (j and k) IFNγ secretion of Teff cells (j) and $CD8^+$ T cells (k) of WT and cKO mice MC38 tumor model injected with anti-IFNγ.

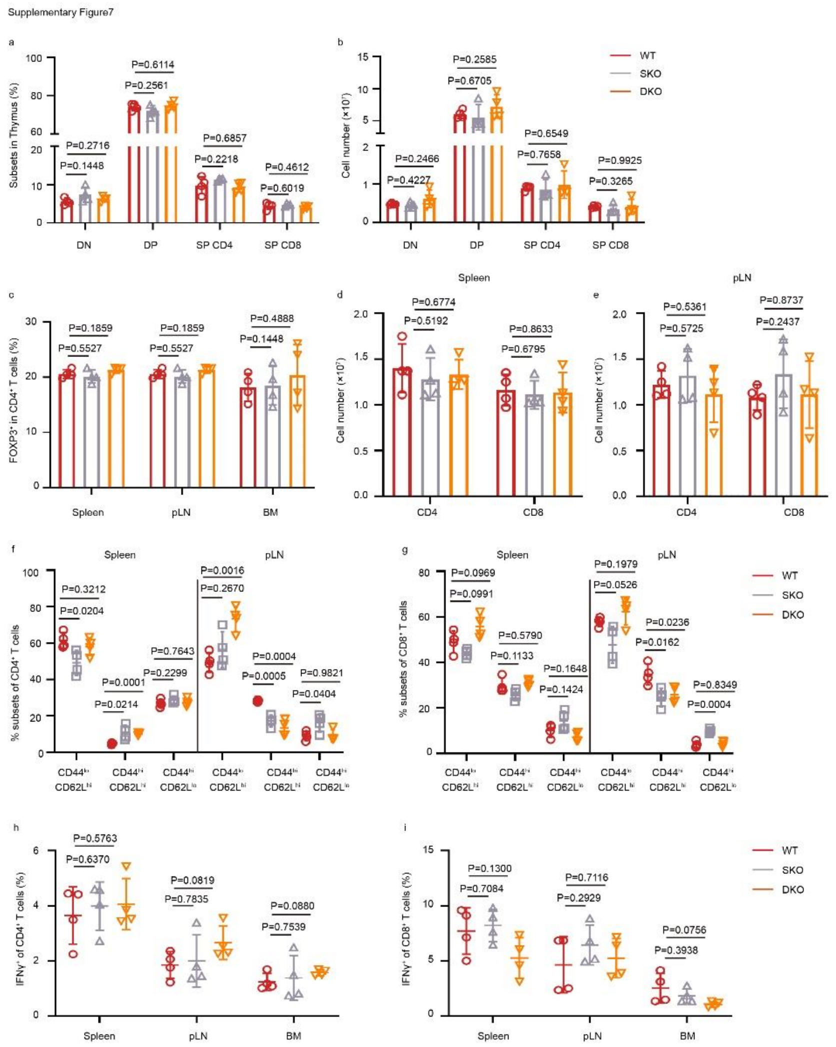

Supplementary Figure 7. IFITM3-STAT1 feedback loop does not influence the T cell homeostasis *in vivo*.

(a) Flow cytometric analysis of the percentage (n=4) and the number (n=4) of $CD4^+$ T cells (SP CD4), $CD8^+$ T cells (SP CD8), $CD4^+CD8^+$ double positive (DP), and $CD4^-CD8^-$double negative (DN) in the thymus from WT, SKO, and DKO mice. (c) Flow cytometric analysis of $FOXP3^+$ Treg cells of $CD4^+$ T cells in spleen and lymph nodes from WT, SKO, and DKO mice (n=4). (d and e) Cell number of $CD4^+$ and $CD8^+$ T cells number in spleen and lymph nodes from WT, SKO, and DKO mice (6-8 weeks) (n=4). (f and g) Flow cytometric analysis of CD44 and CD62L expression in Tconv (f) and $CD8^+$ T cells (g) from WT, SKO, and DKO mice (6-8 weeks) to identify the activation status (n=4). (h and i) Flow cytometric analysis of IFNγ secretion in Tconv (h) and $CD8^+$ T cells (i) from WT, SKO, and DKO mice (6-8 weeks) (n=4). Data are represented as the mean ± SD. *$P < 0.05$, **$P < 0.01$ and ***$P < 0.001$ by 2-tailed Student's t test.

Supplementary Figure8

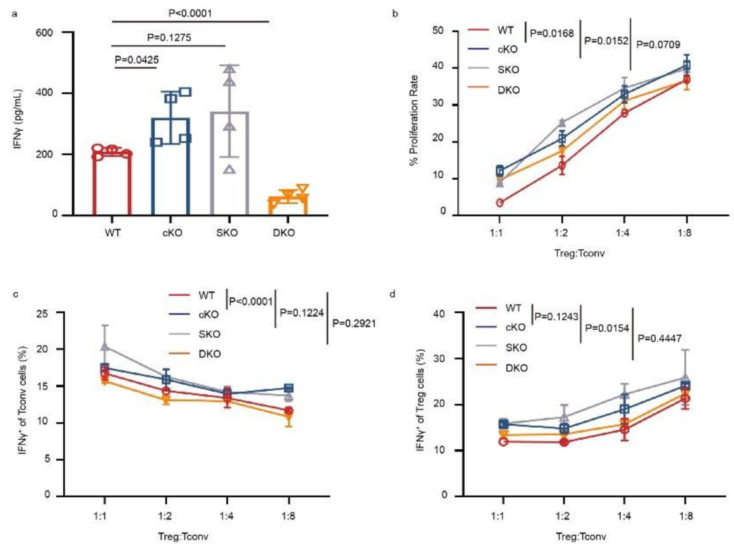

Supplementary Figure 8. IFITM3-STAT1 feedback loop does not influence the T cell homeostasis *in vivo*.

(a) IFNγ content in coculture system of MC38 cells and splenocytes from WT, IKO, SKO, and DKO mice (n=4). (b) The proliferation rate of Tconv cells analyzed by CTV dilution in suppression assay of Treg cells from WT, IKO, SKO, and DKO mice (n=4). (c and d) The IFNγ secretion of Tconv cells (c) and Treg cells (d) in the suppression assay.

Supplementary Figure9

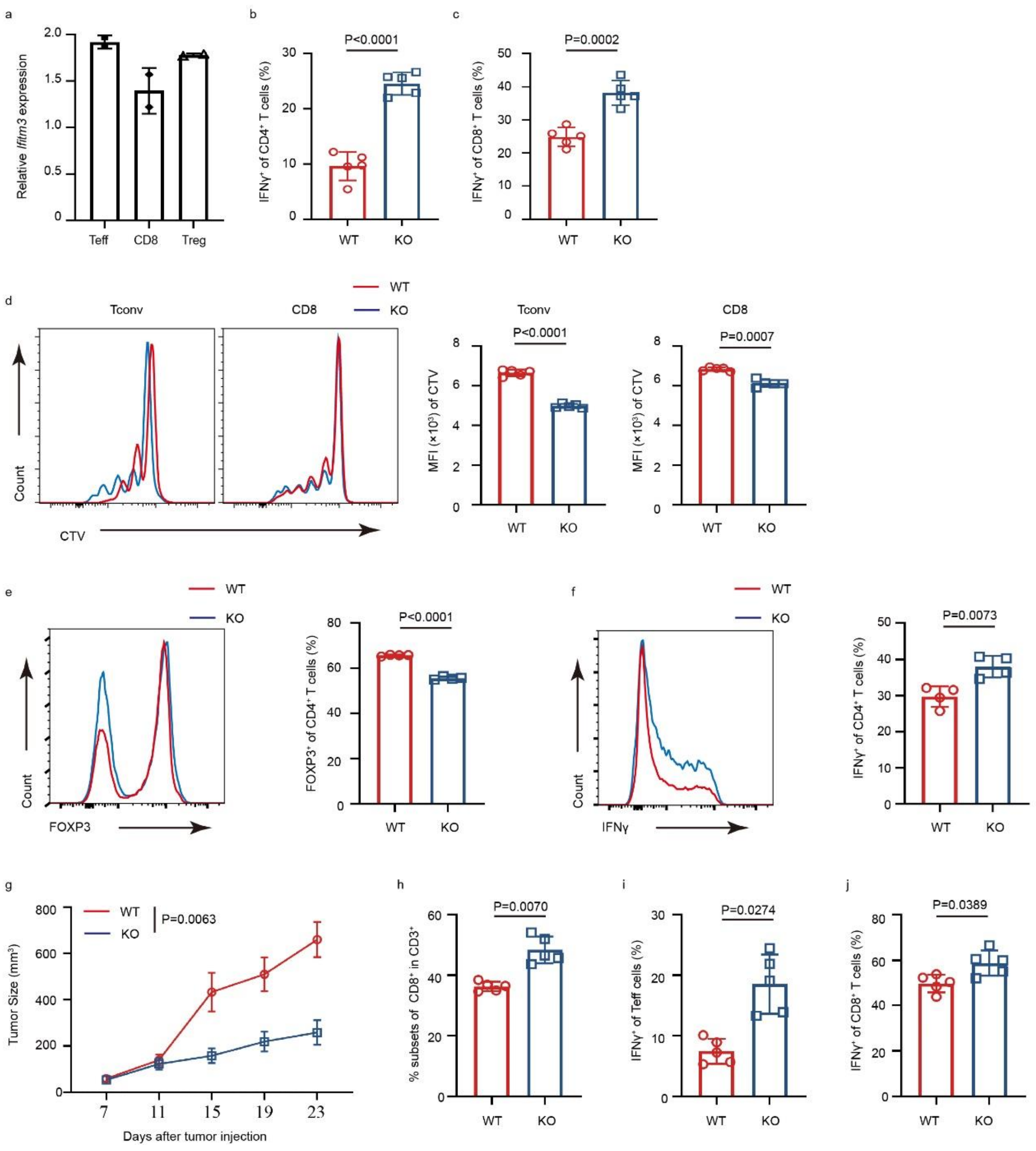

Supplementary Figure 9. IFITM3 also dampens T-cell function and stability in the tumor.

(a) RT-qPCR of *Ifitm3* expression in tumor-infiltrating Teff cells, $CD8^+$ T cells, and Treg cells. (b and c) The IFNγ secretion of $CD4^+$ and $CD8^+$ T cells after TCR stimulation of 24h. (d) Proliferation analyzed by CTV dilution of $CD4^+$ and $CD8^+$ T cells after TCR stimulation of 72h. (e) Flow cytometric analysis of $FOXP3^+$ cells after naïve $CD4^+$ T cells stimulated with anCD3/CD28, mTGFβ, and IL-2. (f) Flow cytometric analysis of Th1 Treg cells after naïve $CD4^+$ T cells stimulated with anCD3/CD28, mIL12, and anti-IL4. (g) Tumor size of WT and KO mice injected s.c. with MC38 murine colon cancer cells (day 23, n=6). (h) Flow cytometric

analysis of FOXP3$^+$ Treg cells in the tumor of WT and KO mice injected s.c. with MC38 murine colon cancer cells (day 23, n=5). (i and j) IFNγ secretion of Teff cells and CD8$^+$ T cells of WT and KO mice injected s.c. with MC38 murine colon cancer cells (day 23, n=5).

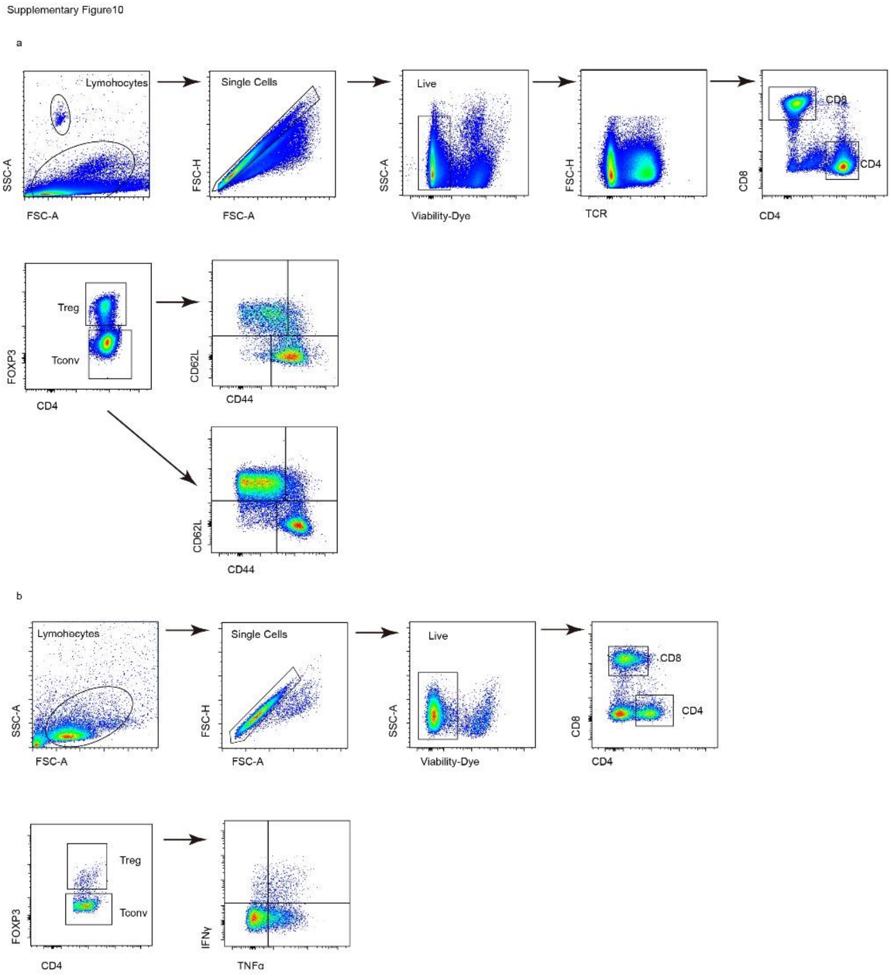

Supplementary Figure 10. Gating strategy of flow cytometry.

(a) Flow cytometry strategy of cell surface staining. (b) Flow cytometry strategy of cytokine staining.

Supplementary Table 1

qPCR primers used in the manuscript.

Gene	Forward Primer	Reverse Primer
mActin	GGCTGTATTCCCCTCCATCG	CCAGTTGGTAACAATGCCATGT
mCtla4	TTTTGTAGCCCTGCTCACTCT	CTGAAGGTTGGGTCACCTGTA
mFoxp3	CCCATCCCCAGGAGTCTTG	ACCATGACTAGGGGCACTGTA
mPdcd1	TAGTGGGTATCCCTGTATTGCT	CTTCTCTCGTCCCTGGAAGTC

mIfitm3	TTCTGCTGCCTGGGCTTCATAG	ACCAAGGTGCTGATGTTCAGGC
mStat1	GCCTCTCATTGTCACCGAAGAAC	TGGCTGACGTTGGAGATCACCA
mTbx21	CAACAACCCCTTTGCCAAAG	TCCCCCAAGCAGTTGACAGT
mIrf1	TCCAAGTCCAGCCGAGACACTA	ACTGCTGTGGTCATCAGGTAGG
mIsg15	CTGCTCCAGTCTAGGGATCG	TTCAGGCACTTAGCAGTGGA
mJak2	GCTACCAGATGGAAACTGTGCG	GCCTCTGTAATGTTGGTGAGATC
hIFITM1	GGCTTCATAGCATTCGCCTACTC	AGATGTTCAGGCACTTGGCGGT
hIFITM3	CTGGGCTTCATAGCATTCGCCT	AGATGTTCAGGCACTTGGCGGT

Supplementary Table2

ChIP-qPCR primers used in the manuscript.

Gene	Forward Primer	Reverse Primer
mPsmb9-ChIP	TCTCCGTGGGGAAGGAAGAAGG	TTCGAGGTCGGCTTTCGGTTTC
mIfi44-ChIP	AAAAGCCTACTGAAGTTGGCTCGC	CTGTCTCAGTGACCAAGCCTGTAG
mIfitm3-ChIP(-203 to -70bp)	GGAACAGTGAGGTTCTGCAGCTG	TGTGAATCAGGGGAACTTCCAGGC
mIfitm3-ChIP(-23 to +109bp)	TTAGATCCCCGCCCCTCTGAAC	AGCACTGAAGGGCAAAGGATGGAG
