# Supplementary material for: FOXP3+ regulatory T cell perturbation mediated by the IFNγ-STAT1-IFITM3 feedback loop is essential for anti-tumor immunity
Source: Nat Commun. 2024 Jan 2;15:122. doi: 10.1038/s41467-023-44391-9 (PMC10761945; doi:10.1038/s41467-023-44391-9)
Supplement: Supplementary file 3 — Reporting Summary [file 41467_2023_44391_MOESM3_ESM.pdf]

nature portfolio

Corresponding author(s): Fan Pan, Hecheng Li, and Bin Li

Last updated by author(s): Nov 18, 2023

Reporting Summary

Nature Portfolio wishes to improve the reproducibility of the work that we publish. This form provides structure for consistency and transparency in reporting. For further information on Nature Portfolio policies, see our Editorial Policies and the Editorial Policy Checklist.

Statistics

For all statistical analyses, confirm that the following items are present in the figure legend, table legend, main text, or Methods section.

n/a	Confirmed	
☐	☒	The exact sample size (*n*) for each experimental group/condition, given as a discrete number and unit of measurement
☐	☒	A statement on whether measurements were taken from distinct samples or whether the same sample was measured repeatedly
☐	☒	The statistical test(s) used AND whether they are one- or two-sided<br>*Only common tests should be described solely by name; describe more complex techniques in the Methods section.*
☒	☐	A description of all covariates tested
☐	☒	A description of any assumptions or corrections, such as tests of normality and adjustment for multiple comparisons
☐	☒	A full description of the statistical parameters including central tendency (e.g. means) or other basic estimates (e.g. regression coefficient) AND variation (e.g. standard deviation) or associated estimates of uncertainty (e.g. confidence intervals)
☐	☒	For null hypothesis testing, the test statistic (e.g. *F*, *t*, *r*) with confidence intervals, effect sizes, degrees of freedom and *P* value noted<br>*Give P values as exact values whenever suitable.*
☒	☐	For Bayesian analysis, information on the choice of priors and Markov chain Monte Carlo settings
☒	☐	For hierarchical and complex designs, identification of the appropriate level for tests and full reporting of outcomes
☒	☐	Estimates of effect sizes (e.g. Cohen's *d*, Pearson's *r*), indicating how they were calculated

Our web collection on statistics for biologists contains articles on many of the points above.

Software and code

Policy information about availability of computer code

Data collection	Flow cytometry data were collected from LSRII (BD) flow cytometry,RT-qPCR data were collected from QuantStudio R7 Flex T-qPCR System (ThermoFisher),RNA-sequencing data were collected from Low-input mRNA Library Preparation (DNBSEQ) BGI-NGS-JK-RNA-007 A0, Western Bloting images were collected from GE Amersham Imager600, ELISA data were collected from Multiskan FC (Thermo Fisher), Immunofluorescence images were acquired from Leica SP8.
Data analysis	Flow Cytometry data were analyzed with FlowJo Version 10.<br>Quantification of WesternBlotting were performed with ImageJ.<br>Statistical analysis were performed with GraphPad Prism8.<br>The RNA-seq data were mapped to Mouse Genome Assembly GRCm38. The analysis of different gene expression were collected from R (version4.2.2). In this process, the DEseq2(version1.38.3), edgeR(version3.40.2), and limma(version3.54.2) packages were used. The DEGs were used to plot heatmaps by the pheatmap(version1.0.12).<br>Immunofluorescence images were analyzed by Leica Application Suite X.

For manuscripts utilizing custom algorithms or software that are central to the research but not yet described in published literature, software must be made available to editors and reviewers. We strongly encourage code deposition in a community repository (e.g. GitHub). See the Nature Portfolio guidelines for submitting code & software for further information.

Data

Policy information about availability of data

All manuscripts must include a data availability statement. This statement should provide the following information, where applicable:

- Accession codes, unique identifiers, or web links for publicly available datasets
- A description of any restrictions on data availability
- For clinical datasets or third party data, please ensure that the statement adheres to our policy

The bulk RNA-seq data generated in this study were transferred to the Gene Expression Omnibus and the transferred files are placed into the processing queue. And the gene expression of COAD and ESCA patients were from TCGA database.

Research involving human participants, their data, or biological material

Policy information about studies with human participants or human data. See also policy information about sex, gender (identity/presentation), and sexual orientation and race, ethnicity and racism.

Reporting on sex and gender	Gender: Female: n=4, Male: n=18. And the gender of the patients is random.
Reporting on race, ethnicity, or other socially relevant groupings	N/A
Population characteristics	Gender: Female: n=4, Male: n=18<br>Age at diagnosis: <70 n=20, >70 n=2<br>Solid tumor tissues and normal tissues were freshly isolated and digested for flow cytometry analysis.The normal tissue in our experiments was the normal tissue from the same tumor patients. Normal tissue adjacent to the tumors was collected from the same patients (5 cm at least away from the tumor edge).
Recruitment	Patient-derived PBMC, ESCA tissues, and matched normal tissues were obtained from Ruijin Hospital, and COAD tissues and matched normal tissues were obtained from Renji Hospital. They are recruited to participate in an Institutional Reviews Board-approved protocol.
Ethics oversight	The study protocol was approved by the ethics committee of Ruijin Hospital (2021-224) and Renji Hospital (KY2022-174-B) complied with all relevant ethics regulations.

Note that full information on the approval of the study protocol must also be provided in the manuscript.

Field-specific reporting

Please select the one below that is the best fit for your research. If you are not sure, read the appropriate sections before making your selection.

☒ Life sciences ☐ Behavioural & social sciences ☐ Ecological, evolutionary & environmental sciences

For a reference copy of the document with all sections, see nature.com/documents/nr-reporting-summary-flat.pdf

Life sciences study design

All studies must disclose on these points even when the disclosure is negative.

Sample size	The number of experiment is not less than two or the sample size is not less than 3to meet the least requirements for statistical analyses.
Data exclusions	No data were excluded from the study.
Replication	The experiments in vitro and in vivo were performed with at least 3 biological replicated or at least two independent repeats. And we were able to repeat the same trends.
Randomization	Littermate mice were randomly grouped for all the experiments. Experimental units including mouse cages, culture wells were randomly organized in this study.
Blinding	Investigators were blinded to group allocation during data collection and analysis.

Reporting for specific materials, systems and methods

We require information from authors about some types of materials, experimental systems and methods used in many studies. Here, indicate whether each material, system or method listed is relevant to your study. If you are not sure if a list item applies to your research, read the appropriate section before selecting a response.

Materials & experimental systems

n/a	Involved in the study
[ ]	[x] Antibodies
[ ]	[x] Eukaryotic cell lines
[x]	[ ] Palaeontology and archaeology
[ ]	[x] Animals and other organisms
[x]	[ ] Clinical data
[x]	[ ] Dual use research of concern
[x]	[ ] Plants

Methods

n/a	Involved in the study
[x]	[ ] ChIP-seq
[ ]	[x] Flow cytometry
[x]	[ ] MRI-based neuroimaging

Antibodies

Antibodies used

anti-Myc(9E10, Santa Cruz, sc-40, 1ug for IP, 1:2000 for WB)
anti-HA(H6908, Sigma-Aldrich, 1ug for IP, 1:2000 for WB)
anti-Flag (F3165, Sigma-Aldrich,1ug for IP, 1:15000 for WB)
anti-LC3B (ab192890, Abcam, 1:1000 for WB)
anti-IFITM3 (11714-1-AP, Proteintech,1:5000 for WB)
anti-STAT1 (14994T, Cell Signaling Technology,1:1000 for WB)
anti-pSTAT1 (Tyr701) (7649T, Cell Signaling Technology,1:1000 for WB)
anti-FOXP3 (14-7979-82, eBioscience,1:3000 for WB)
anti-FOXO1 (2880T, Cell Signaling Technology,1:1000 for WB)
anti-Phospho-FoxO1(Thr24)/FoxO3a (Thr32) (9464T, Cell Signaling Technology,1:1000 for WB)
anti-AKT (60203-2-Ig, Proteintech,1:5000 for WB),
anti-p-AKT (9271T, Cell Signaling Technology,1:1000 for WB)
anti-GAPDH (60004-1-Ig, Proteintech,1:8000 for WB)
anti-β-Actin (66009-1-1g, Proteintech,1:8000 for WB)
anti-LaminB (66095-1-Ig, Proteintech,1:8000 for WB)

Flow cytometry
Viability Dye (65-0865-14, eBioscience,1:1000)
anti-CD4 (45-0042-82, eBioscience, 1:300)
anti-CD8 (563898, BD Pharmingen, 1:300)
anti-CD25 (102008, Biolegend, 1:300)
anti-CD45.2 (109832, Biolegend, 1:300)
anti-CD44 (130-102-606, Miltenyi Biotec, 1:200)
anti-CD62L (20-0621-U100, Tonbo, 1:200)
anti-NK1.1 (11-5941-85, eBioscience, 1:200)
anti-FOXP3 (11-5773-82, eBioscience, 1:300)
anti-IL-17A (130-102-344, Miltenyi Biotec, 1:200)
anti-IFNγ (17-7311-82, eBioscience, 1:200)
anti-Ki67 (25-5698-82, eBioscience, 1:200)
anti-IL10 (12-7101-82, eBioscience, 1:200)
anti-TNFα (12-7321-82, eBioscience, 1:200)
anti-pSTAT1 (666404, Biolegend, 5ug/test)

Validation

All the commercially available antibodies were validated by the manufacturer via immunoblot or IF imaging. All primary antibodies have been validated by WB by using cell lines and over-expressing plasmid.

Eukaryotic cell lines

Policy information about cell lines and Sex and Gender in Research

Cell line source(s)

State the source of each cell line used and the sex of all primary cell lines and cells derived from human participants or vertebrate models.

Authentication

Describe the authentication procedures for each cell line used OR declare that none of the cell lines used were authenticated.

Mycoplasma contamination

Confirm that all cell lines tested negative for mycoplasma contamination OR describe the results of the testing for mycoplasma contamination OR declare that the cell lines were not tested for mycoplasma contamination.

Commonly misidentified lines
(See ICLAC register)

Name any commonly misidentified cell lines used in the study and provide a rationale for their use.

Animals and other research organisms

Policy information about studies involving animals; ARRIVE guidelines recommended for reporting animal research, and Sex and Gender in Research

Laboratory animals	All mouse lines were on the C57BL/6J genetic background. Ifng KO mice were a gift from Gonghua Huang(Guangdong Medical University, China). Ifitm3fl/fl mice and Stat1fl/fl mice were generated by Cyagen Bioscience using a LoxP-targeting system. Foxp3YFP-cre mice and Cd4cre mice were purchased from the Jackson Laboratory (stock number, 016959), which carries an internal ribosome entry site (IRES) and a yellow fluorescent protein (YFP) fused to a codon-optimized Cre recombinase sequence downstream of the internal stop codon of the Foxp3 gene. All mice were allowed free access to food and water and were housed under 12 h light and dark cycles at room temperature (22-26°C) with relative humidity around 40%. All mice were maintained in a specific–pathogen–free (SPF) facility at the Shanghai Jiao Tong University School of Medicine.
Wild animals	The study did not involve wild animals.
Reporting on sex	Male and female mice were sex-matched and used at 6 to 8 weeks of age.
Field-collected samples	The study did not involve samples collected from field.
Ethics oversight	All animal experiments followed the protocols approved by the Institutional Animal Care and Use Committee at the Institute of Shanghai Immunology, School of Medicine, Shanghai Jiao Tong University under protocol number A-2022-031.

Note that full information on the approval of the study protocol must also be provided in the manuscript.

Plants

Seed stocks	*Report on the source of all seed stocks or other plant material used. If applicable, state the seed stock centre and catalogue number. If plant specimens were collected from the field, describe the collection location, date and sampling procedures.*
Novel plant genotypes	*Describe the methods by which all novel plant genotypes were produced. This includes those generated by transgenic approaches, gene editing, chemical/radiation-based mutagenesis and hybridization. For transgenic lines, describe the transformation method, the number of independent lines analyzed and the generation upon which experiments were performed. For gene-edited lines, describe the editor used, the endogenous sequence targeted for editing, the targeting guide RNA sequence (if applicable) and how the editor was applied.*
Authentication	*Describe any authentication procedures for each seed stock used or novel genotype generated. Describe any experiments used to assess the effect of a mutation and, where applicable, how potential secondary effects (e.g. second site T-DNA insertions, mosiacism, off-target gene editing) were examined.*

Flow Cytometry

Plots

Confirm that:

- [x] The axis labels state the marker and fluorochrome used (e.g. CD4-FITC).
- [x] The axis scales are clearly visible. Include numbers along axes only for bottom left plot of group (a 'group' is an analysis of identical markers).
- [x] All plots are contour plots with outliers or pseudocolor plots.
- [x] A numerical value for number of cells or percentage (with statistics) is provided.

Methodology

Sample preparation	To enrich lymphocytes from different tissues like the lung, liver, colon, and subcutaneous fatty tissue (SAT), visceral adipose tissue (VAT), we first cut the tissue into small pieces. Lung, liver, SAT, and VAT were digested for 30-60 min at 37°C with Collagenase D (1 mg ml−1, Roche), DNaseI (500 ugml−1, Roche), and 10% FBS in RPMI 1640 medium. Colon was digested for 60 min at 37°C with Collagenase VIII (1 mg ml−1, Roche), DNaseI (500 ug ml−1, Roche), and 10% FBS in RPMI 1640 medium. MC38 tumors were digested for 30 min at 37°C with Collagenase IV (1 mg ml−1, Roche), DNaseI (500 ug ml−1, Roche), and 10% FBS in RPMI 1640 medium. Then, digested tissues were strained through 40m cell strainers and subjected to flow cytometric analysis.
Instrument	BD LSRFortessa X20
Software	FlowJo Version 10
Cell population abundance	The cell population abundance was determined by flow cytometry and cell counting.

Gating strategy

In all experiments, populations were gated on FSC/SSC. Dead cells were excluded by using Viability Dye staining. Cell populations were identified as described in figures and figure legends. Positive and negative cells were identified based on clear boundaries between the two populations. And in IFITM3 staining, IgG was used as a control.

☒ Tick this box to confirm that a figure exemplifying the gating strategy is provided in the Supplementary Information.
